# Threat appeals reduce impulsive decision making associated with texting while driving: A behavioral economic approach

**DOI:** 10.1371/journal.pone.0213453

**Published:** 2019-03-07

**Authors:** Yusuke Hayashi, Anne M. Foreman, Jonathan E. Friedel, Oliver Wirth

**Affiliations:** 1 Division of Social Sciences and Education, Pennsylvania State University, Hazleton, Hazleton, Pennsylvania, United States of America; 2 Health Effects Laboratory Division, National Institute for Occupational Safety and Health, Morgantown, West Virginia, United States of America; SWPS University of Social Sciences and Humanities, POLAND

## Abstract

The primary purpose of the present study was to examine the effectiveness of threat appeals in influencing impulsive decision making associated with texting while driving. The participants in the treatment group were exposed to a threatening message about the danger of texting while driving, whereas those in the control group were exposed to a non-threatening message. Following the exposure to either message, the participants completed a delay-discounting task that assessed the degree of impulsive decision making in a hypothetical texting-while-driving scenario. A comparison between the groups revealed that the threat appeals reduced the degree of impulsive decision making associated with texting while driving. In addition, the threat appeals led to greater anticipated regret from texting while driving, less favorable attitudes toward texting while driving, and decreased intentions to text while driving in the future in the treatment group. These results suggest that video-based threat appeals are promising intervention strategies for the public health challenge of texting while driving. Implications from the behavioral economic perspective are discussed.

## Introduction

Distracted driving is defined as driving while attention is drawn away from the driving task to focus on another activity [1]. The form of distraction can be visual (e.g., looking away from the roadway), manual (e.g., taking a hand off the steering wheel and manipulating a device or object), or cognitive (e.g., thinking about something other than driving), and all of these forms of distraction increase the risk of a motor vehicle crash [1]. In the United States, 3,450 people were killed in motor vehicle crashes caused by distracted driving in 2016 [2] and an estimated 391,000 people were injured in 2015 [3]. The total economic costs of distraction-related motor vehicle crashes in the United States were estimated to be greater than $40 billion in 2010 [4].

Texting while driving, a major form of distracted driving, is particularly dangerous because it involves all three forms of distraction mentioned above [5]. Despite its danger, texting while driving has been pervasive, with 31.4% and 40.2% of drivers in the United States reporting they have sent and read text messages while driving in the past 30 days, respectively [6]. Texting while driving is even more prevalent in young drivers, with more than 90% of college students reporting that they have texted while driving [7].

To combat this problem, various solutions have been attempted in previous studies, which can be grouped into three categories: legislation, technology-based solutions, and education-based approaches. First, legislation to ban texting while driving for all drivers has been put into place in 47 states and the District of Columbia [8]. The evidence of the effectiveness of laws restricting mobile phone use while driving, however, is mixed [9,10] (see [11] for review). In addition, the laws are very difficult to enforce because texting while driving is an intermittent activity and the drivers may conceal their behavior to avoid being fined [12,13].

Second, with respect to technological interventions, previous research has evaluated whether speech-based text entry methods and/or head-mounted, wearable display system can ameliorate the distraction caused by texting but it was found that drivers are still distracted relative to driving while not texting [14–16]. A smartphone application that blocks incoming messages was shown to reduce novice drivers’ sending text messages while driving, although 15% of the drivers tried to bypass the blocking system [17] (see also [18], who found that 91% of high school students were not interested in using a smartphone application that locks the phone completely while driving).

Third, the public educational campaign, sponsored by both governments (e.g., *U Drive*. *U Text*. *U Pay*., [19]) and industries (e.g., *It Can Wait*, [20]), have brought information on the dangers of texting while driving through various media (see [21] for review). Although a media campaign combined with high-visibility law enforcement show some positive outcomes [22], evidence on the effectiveness of media-only campaigns in reducing texting while driving has yet to be documented [11]. In addition to media campaigns, other educational interventions have also been attempted. These include (a) an educational program at work consisting of a movie, pamphlets/brochures, and “don’t text and drive” signs placed in the parking lot [23]; (b) a package consisting of an anti-texting-while-driving video and daily text message reminder to not text while driving [24]; and (c) a front windshield sticker reminder not to text while driving [25]. These interventions were certainly effective in reducing texting while driving; however, only one of these studies [23] included follow-up data and thus the long-term efficacy of these interventions is unknown.

Taken together, although previous intervention studies have accomplished important steps towards preventing and reducing texting while driving, the varied effectiveness of these approaches calls for further analyses. We believe it is of great significance to shift our attention from “What procedure works?” to “Why does the procedure work?” Such process-level analyses can deepen our understanding of promising intervention strategies, which in turn can contribute to their further development and refinement.

A possible mechanism that appears to underlie the aforementioned interventions is the explicit or implicit arousal of the threat of a motor vehicle crash and/or traffic violation due to texting while driving. *Threat appeals* refer to a persuasive message that attempts to arouse the threat of danger and harm and influences message recipients to adopt recommendations of the message [26,27]. In transportation safety research, threat appeals have been utilized to discourage drivers from various risky driving behaviors, such as texting while driving [28], drinking and driving [29], and speeding [30], and typically include graphic representations of the injury and death due to motor vehicle crashes caused by the risky driving (see [31] for review). A meta-analysis conducted with 13 experimental studies revealed that threat appeals are effective in eliciting fear, yet they do not consistently yield desirable driving outcomes [32]. More recent studies, however, have found threat appeals to be effective in reducing risky driving behaviors. In one study on texting while driving, participants who were exposed to verbal and/or visual cues to death/dying due to texting while driving reported less favorable attitudes toward texting while driving as well as decreased intentions to text while driving [33]. In another study on speeding, drivers who were exposed to a threatening message about speeding showed decreased tendencies to speed in a driving simulator [34].

Although these successful demonstrations of threat appeals are promising, another important challenge toward reducing texting while driving behavior would be to address the inconsistencies among attitudes, intentions, and behaviors [35,36]. To this end, it is essential to consider an important hallmark of texting while driving: Drivers engage in the risky behavior of texting while driving despite being aware of its danger [7]. Generally speaking, drivers, including the ones who text while driving, have negative attitudes toward texting while driving and they are well aware of its danger [7,37–39], yet some of them still report relatively high intentions to text while driving in the future [33]. Even drivers who have low intentions to text while driving may still engage in texting once they are at the wheel. This is because texting while driving is an automatic behavior that can emerge in spite of individual intentions [40,41]. In addition, texting while driving is negatively correlated with impulse control [42–44], another factor that may contribute to intention-behavior discrepancies [45].

We propose that a key to understand potential attitude-behavior and intention-behavior discrepancies is to understand the impulsive decision-making process underlying texting while driving through which drivers choose to text while driving despite knowledge of its dangers. One conceptual framework potentially useful for this purpose is a behavioral economic approach.

Behavioral economics refers to the application of economic concepts and approaches to the study of individuals’ choices and decisions controlled by reinforcement contingencies operating over extended periods of time [46]. From the behavioral economic perspective, impulsive decision making can be operationalized as choice of an immediate yet less favorable outcome over a delayed yet more favorable outcome [47], and such choice can be made deliberately or implicitly/habitually [48]. Along with this conceptualization, texting while driving can be viewed as a trade-off between (a) an immediate reward (i.e., texting message while driving) combined with an increase in the likelihood of a probabilistic punisher (i.e., increased risk of a motor vehicle crash) and (b) a delayed reward (i.e., text message after driving) without a punisher (i.e., no risk of a crash), and it manifests behaviorally as a choice of the immediate reward despite its risk. Note that the trade-off involves multiple outcomes (and thus multiple discounting processes) and the choice is a net function of the multiple processes [49]. This behavioral economic conceptualization that texting while driving is fundamentally an impulsive choice has been supported by previous studies, demonstrating that drivers who frequently text while driving are more vulnerable to impulsive decision making [38,50,51].

From the behavioral economic perspective, threat appeals in the context of texting while driving can be conceptualized as a manipulation to increase (a) the subjective probability of getting into a motor vehicle crash due to texting and/or (b) the subjective aversiveness (or costs) of the motor vehicle crash. This may impact the aforementioned trade-off in the impulsive decision-making process and shift the driver’s choice toward the delayed reward without risk of a crash. If we conceptualize threat appeals in the context of texting while driving in this manner, two apparently distinct areas of research, threat appeals and behavioral economics, can be related in a fruitful way that could potentially produce synergetic effects.

According to the Competing Neurobehavioral Decision Systems (CNDS) theory, a behavioral economic theory employing dual-system models of decision-making (cf. [45,52,53]), decision-making is the product of two completing neurobehavioral systems: (a) the *impulsive system*, which comprises the limbic and paralimbic brain regions (e.g., nucleus accumbens) and values immediate rewards; and (b) the *executive system*, which comprises portions of the prefrontal cortex and may be needed to inhibit the impulsive system and value delayed rewards [54,55]. The theory further posits that the relative activation of these two decision-making systems is associated with delay discounting processes, and by extension, with clinically relevant choices (e.g., drug use).

From the point of view of the CNDS theory, drivers’ choice to text while driving can be characterized by the competition between the two systems: the impulsive system favoring the impulsive choice (i.e., texting while driving with the risk of a motor vehicle crash) and the executive system favoring the self-controlled choice (i.e., texting after arriving at the destination without the risk). If the threat appeals can increase the subjective probability of a motor vehicle crash and/or the subjective aversiveness of the crash, it may shift driver’s decision toward the self-controlled choice by increasing the relative dominance of the executive system. This is consistent with previous studies using neuroimaging techniques that threat appeals increased activation in the medial prefrontal cortex [56–58] that is associated with executive control [59] and decision making about risk and reward [60].

Despite this potential effectiveness, no previous study has examined the effects of threat appeals on the impulsive decision-making process associated with texting while driving. To fill this gap in the literature, the first purpose of the present study was to examine the effectiveness of threat appeals in influencing such impulsive decision making as assessed by the degree of delay discounting—the subjective devaluation of a future reward (i.e., text message) as a function of delay to its receipt [47]. The participants in the treatment group were exposed to a threatening message on the danger of texting while driving delivered by a video clip on a computer, whereas those in the control group were exposed to a non-threatening message. In addition to the degree of impulsive decision making, the present study examined whether the threatening message can alter attitudes toward and intentions of texting while driving. It was hypothesized that the treatment group would show a lower degree of impulsive decision making, less favorable attitudes, and decreased intentions associated with texting while driving.

Although fear is an emotion that is often elicited by threat appeals [32], it is not the only emotion elicited by them [61]. Threat appeals can elicit various other emotions, such as guilt, shame, and regret, and the interplay among these different emotions should be important to determine the effectiveness of threat appeals [34]. One potential emotion that may be relevant to threat appeals for texting while driving is regret. Indeed, Koch (2014) even claimed that threat-appeal interventions influence message recipients via increasing feelings of regret that they would experience for taking (or not taking) the action mentioned in the message [62].

Regret is an aversive cognitive emotion that is experienced when we realize or imagine that our current situation would have been better, if only we had acted differently [63]. Previous research has shown that making individuals aware of future regret (i.e., anticipated regret) reduces undesirable behaviors, such as risky driving, risky sexual behavior, and substance use (see [64,65] for meta-analyses). In addition, previous research has demonstrated that anticipated regret is effective in influencing decision making associated with risky sexual behavior, as measured by delay discounting [66]. Therefore, it is possible that interventions that increase awareness of anticipated regret are effective for reducing texting while driving.

The second purpose of the present study was to evaluate whether (a) video-based threat appeals can induce the emotion of anticipated regret and, if so, (b) levels of anticipated regret are associated with levels of impulsive decision making associated with texting while driving and attitudes toward and intentions of texting while driving. Unlike previous studies that presented the threat of participants’ dying/being injured in a crash caused by texting while driving [28,33], the present study presented threats of killing/injuring someone due to a crash caused by texting while driving, in an attempt to enhance the emotion of regret being elicited. It was hypothesized that participants who were exposed to threats of potentially killing someone due to texting while driving would exhibit greater levels of anticipated regret, and anticipated regret would serve as a mediating variable between the threat appeals and each of the three dependent measures: impulsive decision making, attitude, and intention of texting while driving.

## Materials and methods

### Participants

One hundred forty undergraduate students enrolled in introductory psychology courses at Pennsylvania State University, Hazleton participated. Participants were recruited in two phases and the two cohorts of participants were exposed to slightly different procedures (details below). They were offered course credit for participation. Students who reported that they did not have a valid driving license (*n* = 27) or that they experienced technical difficulties (*n* = 13; details described below) were excluded from the study (i.e., their data were not analyzed). The retained sample was composed of 39 males and 61 females. Their mean age, years of higher education, and years driving were 19.8 (*SD* = 2.4; ranging from 18 to 30), 1.8 (*SD* = 1.2; ranging from 1 to 7), and 3.1 (*SD* = 2.3; ranging from 0 to 14). The Institutional Review Board at the Pennsylvania State University approved the study protocol.

### Procedure

All surveys were hosted online by Qualtrics (Provo, UT). Participants received an email through the Qualtrics website that contained a link to the online survey. After the participants agreed to participate in the study by clicking “I agree to participate” button as a part of the informed consent process, they completed a demographic questionnaire that asked their age, gender, years of higher education, whether they have a valid driver’s license, and years driving. They then watched a short video clip, completed a hypothetical delay discounting task, and completed a questionnaire that consisted of questions concerning texting while driving. Participants were randomly assigned to either the treatment or the control group, through the Qualtrics’ randomizer function that roughly equated the number of participants across the groups.

#### Video clip

Before any video clip, a YouTube video embedded in the Qualtrics page, was presented, participants were told that they would watch a short video, and were instructed to turn their audio on. For the second cohort of participants, a test video clip was added because many participants in the first cohort reported that they experienced technical difficulties (details descried below). The test video clip presented a non-driving scene and the participants were asked whether they could watch the video clip and listen to the audio before they watched a driving-related video clip.

On the next screen, another video clip was set up to start playing automatically by the YouTube’s auto-play function. While the video was playing, no button to proceed to the next screen was presented. After 1 min, the screen advanced automatically by the Qualtrics’ auto-advance function. Participants in the treatment group watched a video that features a young female driver in a car (available at https://www.youtube.com/watch?v=l7ljxDjwDjU). The driver receives a text message, replies to it, and almost hits the car coming from the other direction. Then, a hypothetical scene starts in which time stops and the driver can speak with the other driver. In the conversation, the driver knows that two young children will be involved in the crash to occur and apologizes for causing the crash due to texting, indicating a sense of regret. At the end of the video, the hypothetical scene ends and time resumes. The video ends when two cars smash into each other. Participants in the control group watched a video (car commercial) that features a man sitting in a car, which faces a bull standing in the middle of the road (available at https://www.youtube.com/watch?v=FoGGDKV88Fg). In the video, the car mostly remains stopped on the road, while the man talks to himself about the bull. At the end of the video, the man slowly turns the car around and drives in the opposite direction. The video contains no texting while driving nor any negative consequence due to texting while driving.

On completion of each video, participants were asked “Did you experience any technical problems (e.g., video not shown, no audio, etc.) in watching the video?” with the option to select either *Yes* or *No*. For the first cohort, 9 out of 36 participants reported they had technical issues. The rate of technical difficulties decreased to 4 out of 77 for the second cohort when the aforementioned test video clip was added. The data from these 13 participants who reported technical issues (11.5% of the total sample) were excluded from analyses.

#### Delay discounting task

After watching the video assigned to their group, participants in both groups completed the same delay-discounting task adopted from [51] that assessed the degree of impulsive decision making associated with texting while driving. Using visual analog scales (VAS), participants rated their likelihood of replying to a text message while driving versus waiting to reply until arriving at a destination. The task presented the following hypothetical scenario:

Imagine that your significant other (or your best friend) has just sent a text message saying “text me asap” while you are driving at 40 mph. You will arrive at the destination in [delay]. Please rate how likely you are to reply now versus waiting for [delay].

The VAS was located immediately below the instruction. It was a horizontal line labeled from 0 to 100 in increments of 10 with the descriptive anchors, *definitely reply now* (far left) and *definitely wait* (far right). The default position of a slider of the VAS was 50, and the participants clicked on the slider and moved it across the horizontal line to indicate their likelihood of waiting until the destination. The VAS was presented six times, each time on a different screen with the only difference being the delay to the destination (30 s, 5 min, 15 min, 30 min, 1 h, and 2 h presented in this ascending order).

#### Questionnaires

Following the completion of the delay discounting task, the participants completed questionnaires that measures the following five categories of information associated with texting while driving (hereafter TWD): (a) the degree to which participants would feel regret if they text while driving in the future (hereafter referred to as *regret*; Cronbach’s *α* = .958); (b) attitudes towards texting while driving (*attitude*; *α* = .869); (c) intentions of engaging in texting while driving in the future (*intention*; *α* = .862); (d) the degree to which they feel they can control their behavior of texting while driving (*perceived efficacy*; *α* = .774); and (e) self-reported frequency of texting while driving in the past (*past TWD frequency*; *α* = .948). The participants also completed a questionnaire that assessed the degree to which the video made the participants think of potentially killing someone due to texting while driving (*perceived threat*; *α* = .950). Note that we chose to measure the level of the perceived threat of potentially killing someone, rather than the fear of killing someone, because the level of threat as a stimulus would better represent the effectiveness of the independent variable (the video clip) than the level of fear as a response to the stimulus, which could vary across individuals [67]. Also, we focused on regret as an emotion aroused by threat appeals, as mentioned previously. See S1 Appendix for all the questions presented. Means across items were calculated to make the scores directly comparable across these questionnaires (i.e., all scores rage from 1 to 7).

### Data analysis

As a descriptive, non-theoretical measure of delay discounting, the area under the curve (AUC) was calculated at the individual level according to the method described by [68]. AUC provides a single index of the degree of discounting that is standardized to range from 0 (completely discounted) to 1 (not discounted at all). We chose to use AUC because this standardized score allows for comparisons across studies, regardless of the units being employed.

With respect to statistical analyses, gender was analyzed as a function of the groups with a chi-square test. Other continuous variables were also analyzed as a function of the groups with an independent sample *t*-test. In addition, an analysis of covariance (ANCOVA) was performed on the four TWD-related dependent measures (AUC, regret, attitude, and intention) with age and past TWD frequency as covariates. Age and past TWD frequency were chosen because of their significant correlations with the AUC measure (see below). Correlational analyses were performed by calculating Pearson correlation coefficients. Simple mediation analyses were performed with the PROCESS macro (Version 3.2) developed by [69], utilizing a 5000-sample bootstrapping procedure. In these analyses, threat appeals served as the independent variable, regret served as the mediator, age and past TWD frequency served as covariates, and AUC, attitude, and intention served as the dependent variables. The significance of indirect (mediational) effect was assessed with percentile bootstrap confidence intervals (i.e., the upper and lower bound of the intervals not containing zero) [69]. All assumptions of the statistical tests were met with an exception for normality of age, years of higher education, and years driving. Nevertheless, we deemed the *t*-test appropriate for these variables because previous research has well established that the *t*-test is fairly robust for the violation of the normality assumption [70–72]. All statistical analyses were performed with SPSS Version 25. The statistical significance level was set at .05.

## Results

Table 1 shows the demographic characteristics and perceived efficacy and threat for both groups. No significant differences among groups were found for gender, *χ*^*2*^(1) = .00, *p* = .964; age, *t*(98) = -.83, *p* = .411; years of higher education, *t*(98) = -.45, *p* = .656; years driving, *t*(98) = -.45, *p* = .652; past TWD frequency, *t*(98) = .33, *p* = .740; or perceived efficacy, *t*(98) = 1.00, *p* = .321. With respect to the perceived threat of potentially killing someone due to texting while driving (i.e., manipulation check), there was a significant difference between groups, *t*(98) = -7.01, *p* < .001.

**Table 1:** Demographic characteristics and perceived efficacy and threat for the control and treatment groups.

Characteristics	Control	Treatment
Gender		
Male	20	19
Female	31	30
Age in years	19.6 (2.2)	20.0 (2.5)
Years of higher education	1.8 (1.3)	1.9 (1.2)
Years driving	3.0 (2.0)	3.2 (2.5)
Past TWD frequency	2.9 (1.6)	2.8 (1.8)
Perceived efficacy	6.5 (1.1)	6.2 (1.5)
Perceived threat***	2.9 (1.9)	5.4 (1.7)

*Note*. The numbers are means (and standard deviations) except for gender. TWD = Texting while driving.

***p < .001.

**Fig 1:**
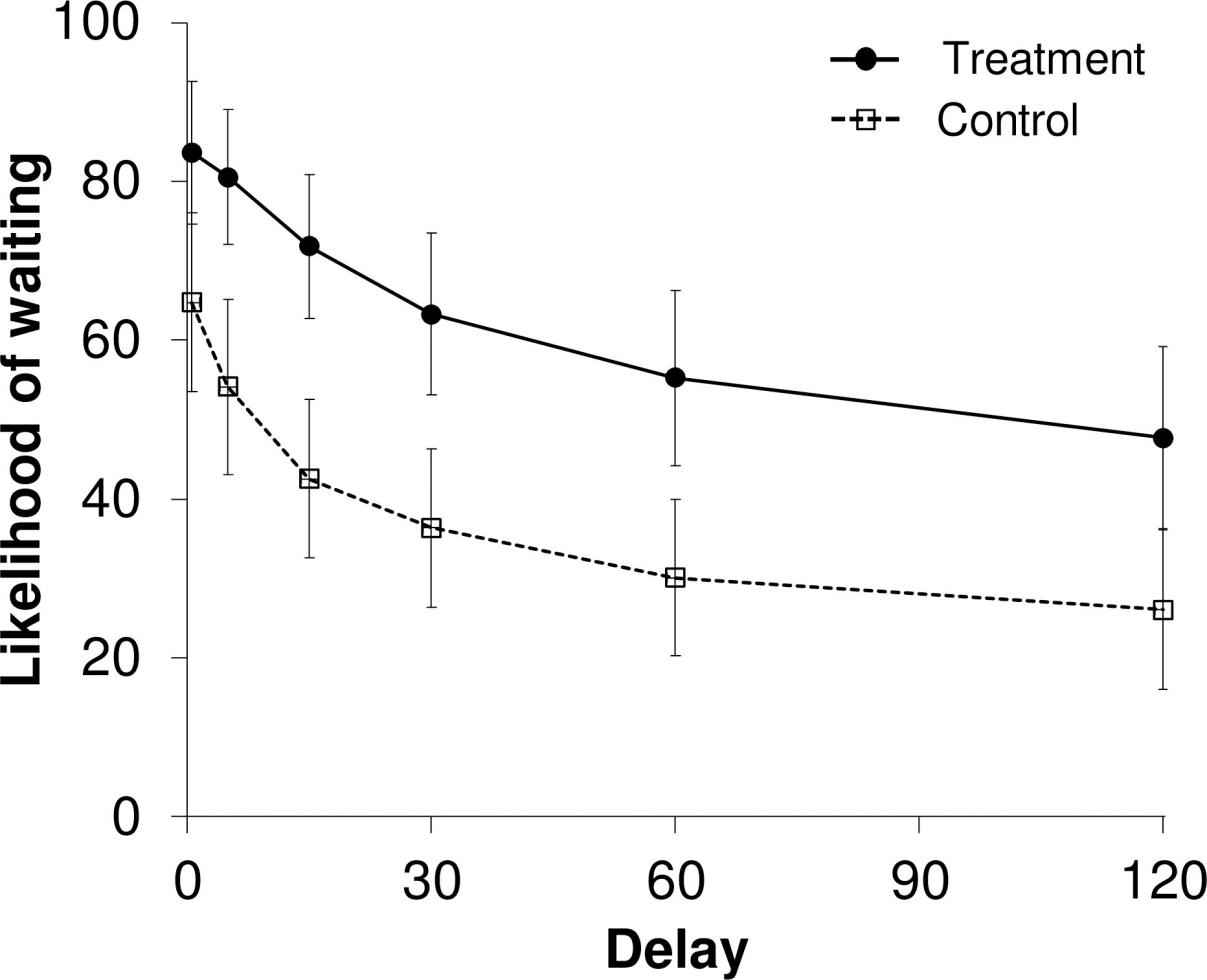
Mean likelihood of waiting to reply to a text message as a function of delay to the destination for the treatment and control groups. Error bars represent 95% confidence intervals.

Fig 1 shows the mean likelihood of waiting to reply to a text message for both groups. The likelihood of waiting decreased as a function of delay to the destination for both groups, suggesting that the value of social interaction from texting is subject to delay discounting. The rates of delay discounting varied greatly between the two groups: The visual analysis of the figure indicates that the treatment group showed less steep discounting (i.e., less impulsive decision making). The results of an independent sample *t*-test based on AUC data calculated from individual participants revealed the AUC value was significantly higher for the treatment group (*M* = 0.59, *SD* = 0.33) than for the control group (*M* = 0.33, *SD* = 0.33), *t*(98) = 3.81, *p* < .001, Cohen's *d* = .76.

**Fig 2:**
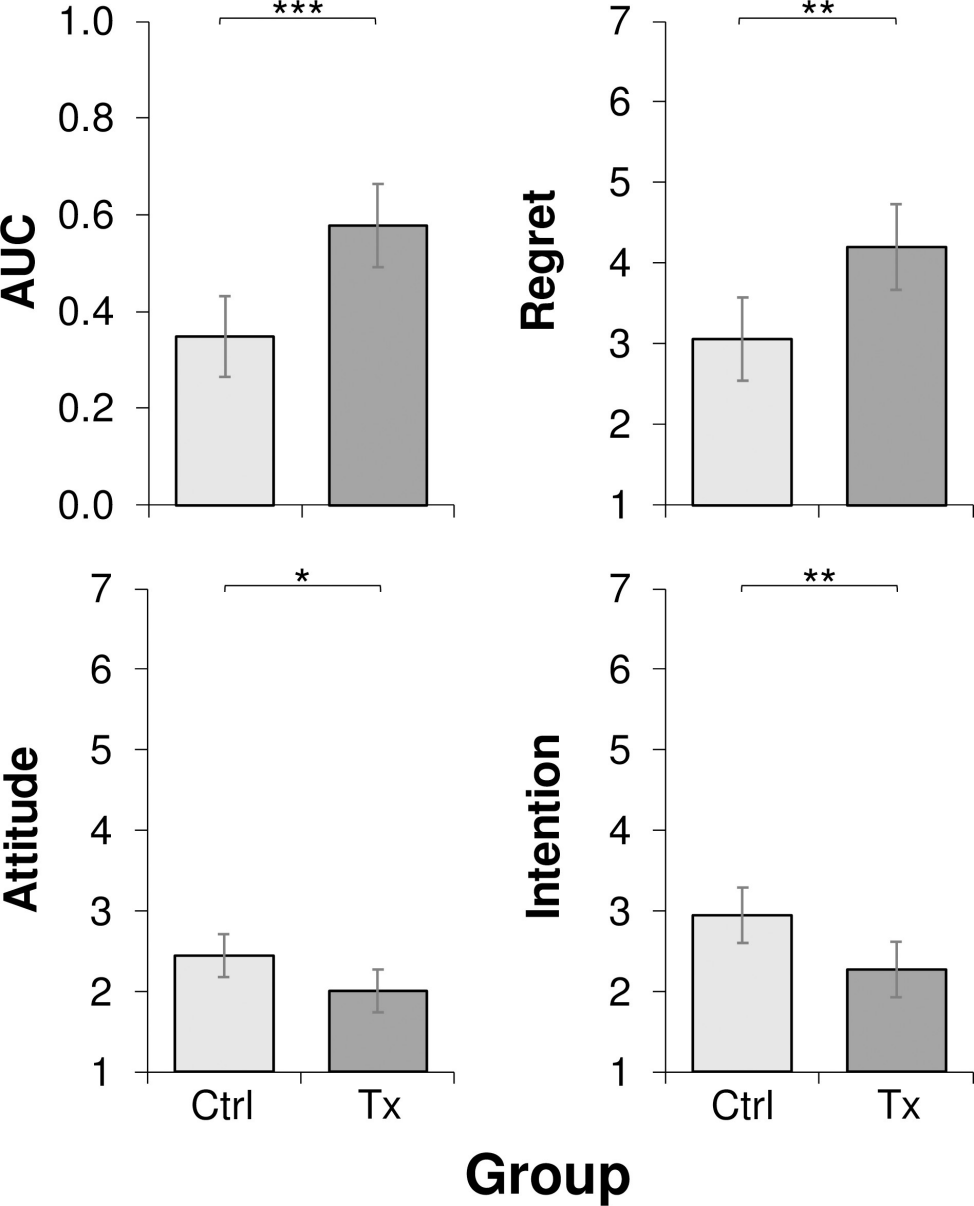
Adjusted means for area under the curve (AUC), anticipated regret due to texting while driving (regret), attitudes toward texting while driving (attitude), and intention of texting while driving in the future (intention) for the treatment (Tx) and control (Ctrl) groups. Error bars represent 95% confidence intervals. *p < .05. **p < .01. ***p < .001.

Fig 2 shows adjusted means of AUC, regret, attitude, and intention for both groups. After adjustment for age and past TWD frequency, the results of ANCOVA’s revealed significant differences between groups on AUC, *F*(1, 96) = 14.55, *p* < .001, η_p_^2^ = .132; regret, *F*(1, 96) = 9.28, *p* = .003, η_p_^2^ = .088; attitude, *F*(1, 96) = 5.25, *p* = .024, η_p_^2^ = .052; and intention, *F*(1, 96) = 7.33, *p* = .008, η_p_^2^ = .071.

Table 2 shows Pearson correlation coefficients among the demographic characteristics, perceived efficacy and threat, and the TWD-related measures. First, age was significantly correlated with AUC and attitude (*p*’s < .05), and past TWD frequency was significantly correlated with AUC, regret, attitude, and intention (*p*’s < .05). This is why age and past TWD frequency were controlled for when ANCOVA was performed. Second, AUC, regret, attitude, and intention were significantly correlated with each other (all *p*’s < .05).

**Table 2:** Pearson correlation coefficients among demographic characteristics, perceived efficacy and threat, and TWD-Related measures.

	1	2	3	4	5	6	7		8	9	10	11
1. Age	-											
2. Gender (F = 0)	.09**	-										
3. Years of education	.53**	.00**	-									
4. Years driving	.81**	.13**	.51**	-								
5. Past TWD frequency	-.13**	-.02**	-.01**	-.01	-							
6. Perceived Efficacy	.15**	.29**	.03**	.18	-.08	-						
7. Perceived Threat	.01**	-.25**	.07**	.05	.00	-.20*		-				
8. AUC	.27**	-.05**	.08**	.13	-.34**	-.08*	.39**		-			
9. Regret	.09**	-.15**	.10**	.11	-.35**	-.04*	.40**		.40**	-		
10. Attitude	.24**	.10**	-.14**	-.13	.32**	-.01*	-.17**		-.39**	-.53**	-	
11. Intention	-.14**	.18**	-.06**	-.02	.58**	-.12*	-.20**		-.40**	-.56**	.58**	-

*Note*. TWD = texting while driving. AUC = Area under the Curve.

*p < .05.

**p < .01.

**Fig 3:**
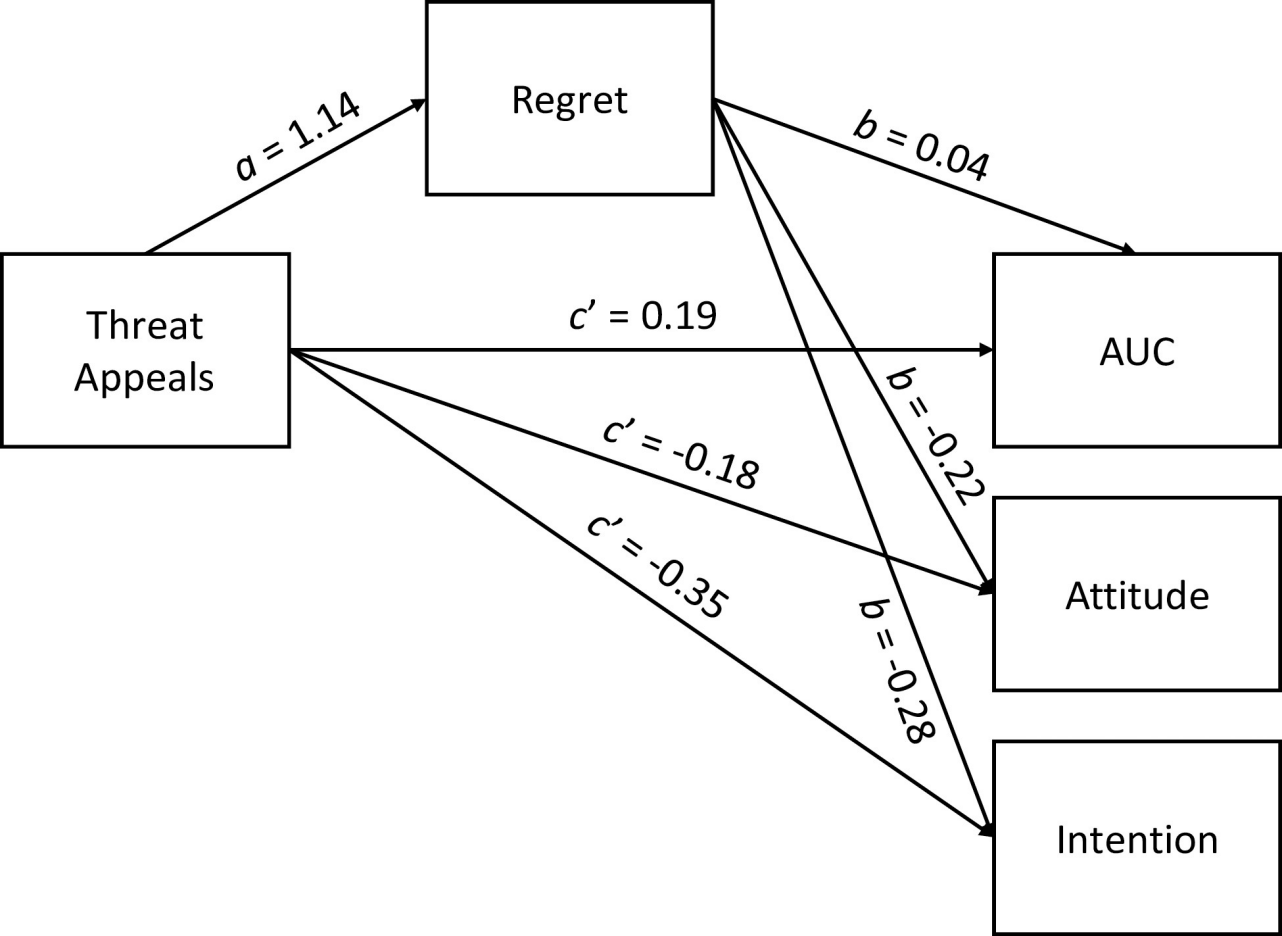
Diagram of the mediator models with regret as potential mediators between threat appeals and AUC, attitude, and intention. *ab* = indirect effect. *c’* = direct effect. The values indicate unstandardized coefficients.

Fig 3 shows the path diagram of the mediation models. To determine if there were significant indirect effects, we examined the bootstrapped 95% confidence intervals. None of the confidence intervals contained 0, indicating significant indirect effects of threat appeals through regret on AUC: *ab* = 0.04, 95% confidence interval (CI) [0.003, 0.101]; attitude: *ab* = -0.25, 95% CI [-0.470, -0.073]; and intention: *ab* = -0.32, 95% CI [-0.634, -0.101]. The direct effect of threat appeals was significant on AUC: *c’* = 0.19, *t* = 3.03, *p* = .003; but not on attitude: *c’* = -0.18, *t* = -1.02, *p* = .308; and intention: *c’* = -0.35, *t* = -1.47, *p* = .144. The proportion of the effect mediated by regret (indirect effect divided by total effect) was 0.19 for AUC, 0.58 for attitude, and 0.48 for intention.

## Discussion

The first purpose of the present study was to examine the effectiveness of threat appeals in influencing impulsive decision making associated with texting while driving. The participants in the treatment group were exposed to the threatening message about the danger of texting while driving, whereas those in the control group were exposed to a non-threatening message. The results show that the threat appeals reduced the degree of impulsive decision making associated with texting while driving, as assessed by the delay discounting task. The threat appeals also improved attitudes toward texting while driving and reduced intentions of engaging in texting while driving in the future.

The second purpose of the present study was to evaluate two facets of anticipated regret and texting while driving. First, this study evaluated whether anticipated regret can be induced by a threat of potentially killing someone due to a crash caused by texting while driving. The results showed that the threat appeals effectively induced anticipated regret. This study also evaluated whether anticipated regret can mediate the relationship between threat appeals and impulsive decision making, attitudes, and intentions in relation to texting while driving. The results show that anticipated regret partially mediates the relationship between threat appeals and impulsive decision making, indicating that threat appeals influence impulsive decision making both directly and through anticipated regret. The results also show that anticipated regret mediated the relationship between threat appeals and attitudes and intentions in relation to texting while driving, indicating that threat appeals influence attitudes and intentions through anticipated regret.

The present finding supports the notion that threat appeals can evoke emotions other than fear [34] and is consistent with Koch’s (2014) claim that interventions utilizing threat appeals may work via increasing anticipated regret [62]. With respect to transportation safety, previous correlational studies have shown that anticipated regret is negatively associated with intentions to drive recklessly (e.g., cutting in suddenly) [73,74], intentions to speed [75–78], and intentions to text while driving [79,80]. To our knowledge, there is only one experimental study in which the level of anticipated regret was manipulated [81]. In their study, an anti-speeding video failed to influence intentions about future speeding, and the authors reasoned that the video did not elicit sufficient regret based on the results of the manipulation check. The present study, therefore, is the first successful demonstration in the transportation safety literature in which the level of anticipated regret was successfully manipulated and intentions to text while driving were reduced. Because fear-arousing threat appeals are occasionally observed to *increase* risky driving behaviors (see [32] for review), a promising approach may be to base threat appeals on other emotions, such as anticipated regret. Future research should replicate the present finding to further examine the effectiveness of anticipated regret for texting while driving as well as evaluate the relative contribution of fear and anticipate regret on reducing texting while driving.

### Perceived threat and efficacy as critical factors in threat appeals

The current theoretical perspectives on threat appeals emphasize the role of two constructs—*perceived threat* and *perceived efficacy*—in explaining how threat appeals lead to the changes in attitudes and intentions in message recipients [34]. According to the *Extended Parallel Process Model* (EPPM, [82,83]), threat appeals are effective only when message recipients perceive (a) that the susceptibility and the severity of the threat are high (perceived threat), and (b) that they are capable of performing recommended actions that will result in desirable consequences (perceived efficacy). Message recipients first appraise the susceptibility and the severity of the threat elicited by the message. If perceived threat is sufficiently high (but not too high [84]), the levels of perceived efficacy will determine the action to deal with the threat [85]. If high efficacy is perceived, message recipients avert the threat through self-protective changes in behavior (i.e., stop texting while driving). If, instead, low efficacy is perceived, message recipients reduce the fear, not the threat, through maladaptive coping mechanisms, such as rationalization (i.e., believe they can text while driving safely), and thus no behavioral change should be produced.

The results of the previous and present studies are consistent with the EPPM’s predictions. In explaining the *boomerang effect*, in which message recipients reacted against the message, Lennon et al. (2010) reasoned that the threat appeals used in their study did not elicit high levels of threat of texting while driving, which resulted in increased intentions to text while driving [28]. In contrast, in both Kareklas and Muehling (2014) and the present study, in which intentions to text while driving were decreased, the threat appeals effectively elicited the high levels of threat of their own death and killing someone due to texting while driving, respectively, as evidenced by the scores of the manipulation check questions [33]. With respect to perceived efficacy that determines the effectiveness of threat appeals when high threat is perceived [85], it is said that the efficacy is typically high in texting while driving because drivers can choose not to text while driving and doing so should result in the desirable outcome (i.e., avoiding a crash due to texting while driving) [33]. The present study, however, empirically confirmed this claim by demonstrating that drivers perceive strong control over the choice to engage in texting while driving (see Table 1).

It is widely considered that perceived efficacy, particularly response efficacy—the degree to which a recommended action will result in desirable consequences, is one of the critical characteristics in message persuasiveness [86]. Along this line, it is important to note that, although the threat appeals used in the present study were effective, they did not convey any information on coping strategies that allowed drivers to avoid texting while driving and how effective such strategies are (e.g., the use of smartphone applications to automatically reply to incoming text messages). Further examination of the effectiveness of a threat-appeal message that focuses on response efficacy should be an important avenue for future research.

### Limitations

There are four limitations that are noteworthy. First, the present study assessed the immediate (i.e. short-term) impact of the threat-appeal message, and we did not measure its enduring effects. It is advisable for future research to collect follow-up data to evaluate whether positive effects of the threat appeals can be maintained. Second, we did not employ attention check questions on the video. Although the results of the manipulation check question indicate that the treatment group had significantly higher perceived threat, this does not guarantee that participants watched the video clip in a strict sense. Because the information from a message has to be processed properly to persuade a recipient and the capacity to process information is limited [87], future studies are advised to include attention check questions. Third, the decision-making task in the present study was hypothetical and the effects of the threat appeals on the actual texting while driving behavior were not evaluated. It is important to note, however, that previous studies using monetary rewards have established that hypothetical and real outcomes produce similar results [88–94]. As it is unethical to let participants engage in situ texting while driving, the use of the hypothetical task in the present study should be acceptable, at least to evaluate the potential effectiveness of the threat appeals. Finally, the sample of the present study was relatively small and was exclusively composed of college students. Although a meta-analysis concluded that individual differences have little influence on how message recipients respond to threat appeals [95], it is still advisable for future research to employ a more diverse and larger sample to assess the generality of the present findings.

### Future research and conclusion

In addition to providing the theoretical foundation of the present study, the CNDS theory makes several predictions about how to modulate texting while driving that can guide future research. For example, any manipulation that increases the relative dominance of the impulsive system, such as presenting cues associated with the reward of interest [96] or cues associated with a strong reward but not related to the reward of interest [97,98], should be associated with increased occurrence of the impulsive choice of texting while driving. Along this line, assessing the degree of valuation of texting while driving [99] as an assessment of the strength of the impulsive system may be useful (see [46] for the theoretical foundation). Another manipulation of interest would be one that increases the relative dominance of the executive system, which includes threat appeals or episodic future thinking [100,101], executive function training [102], mindfulness-based interventions [103,104], and contingency management [105,106]. All of these should be important avenues for future research, which would lead to the development and refinement of the effective prevention and intervention strategies for the public health challenge of texting while driving.

The present study was undertaken with a specific focus on providing additional insights regarding the use of persuasive communication techniques that may successfully alter and curb the incidence of texting while driving. Given media-delivered educational campaigns continue to be one of the viable vehicles for this purpose, we examined the effectiveness of the video-based threat appeals that were designed to elicit an emotion of anticipated regret due to texting while driving. The threat appeals successfully reduced impulsive decision making associated with texting while driving, generated less favorable attitudes toward texting while driving, and decreased intentions to text while driving in the future. The present study contributes to the transportation safety literature by demonstrating that threat appeals with high perceived efficacy can be a valuable tool for reducing the public health challenge of texting while driving. In addition, the present study demonstrated the fruitfulness of combining two paradigms, threat appeals and behavioral economics, which could lead to effective prevention and intervention strategies for various societal problems.

## Supporting information

S1 Appendix Questionnaires for TWD-related measures and manipulation check. (DOCX)

S1 Dataset Raw data used for the analyses. (CSV)

## References

[1] National Highway Traffic Safety Administration. Distraction by cell phones and texting [Internet]. 2014. Available: http://www.distraction.gov/downloads/pdfs/Distraction-Cell-Phones-Texting.pdf

[2] National Highway Traffic Safety Administration. Distracted driving 2016 [Internet]. 2018. Available: https://crashstats.nhtsa.dot.gov/Api/Public/ViewPublication/812517

[3] National Highway Traffic Safety Administration. Distracted driving 2015 [Internet]. 2017. Available: https://crashstats.nhtsa.dot.gov/Api/Public/ViewPublication/812381

[4] Blincoe LJ, Miller TR, Zaloshnja E, Lawrence BA. The economic and societal impact of motor vehicle crashes, 2010. (Revised). Report No. DOT HS 812 013. Washington, DC: National Highway Traffic Safety Administration; 2015.

[5] Sherin KM, Lowe AL, Harvey BJ, Leiva DF, Malik A, Matthews S, et al Preventing texting while driving: A statement of the American College of Preventive Medicine. Am J Prev Med. 2014;47: 681–688. 10.1016/j.amepre.2014.07.004 25217096

[6] AAA Foundation for Traffic Safety. 2016 traffic safety culture index [Internet]. 2017. Available: https://www.aaafoundation.org/sites/default/files/2016TrafficSafetyCultureIndexReportandCover_0.pdf

[7] Atchley P, Atwood S, Boulton A. The choice to text and drive in younger drivers: Behavior may shape attitude. Accid Anal Prev. 2011;43: 134–142. 10.1016/j.aap.2010.08.003 21094307

[8] Governors Highway Safety Association. Distracted driving laws by state [Internet]. 2018. Available: https://www.ghsa.org/sites/default/files/2018-04/DistractedDrivingLawChart_Apr18.pdf

[9] Ehsani JP, Bingham CR, Ionides E, Childers D. The impact of Michigan’s text messaging restriction on motor vehicle crashes. J Adolesc Health. 2014;54: S68–S74. 10.1016/j.jadohealth.2014.01.003 24759444

[10] Ferdinand AO, Menachemi N, Blackburn JL, Sen B, Nelson L, Morrisey M. The impact of texting bans on motor vehicle crash–related hospitalizations. Am J Public Health. 2015;105: 859–865. 10.2105/AJPH.2014.302537 25790409 PMC4386499

[11] Delgado MK, Wanner KJ, McDonald C. Adolescent cellphone use while driving: An overview of the literature and promising future directions for prevention. Media Commun. 2016;4: 79–89. 10.17645/mac.v4i3.536 27695663 PMC5041591

[12] Gauld CS, Lewis I, White KM. Concealing their communication: Exploring psychosocial predictors of young drivers’ intentions and engagement in concealed texting. Accid Anal Prev. 2014;62: 285–293. 10.1016/j.aap.2013.10.016 24211560

[13] Gilbert JE, Martin AM, Eugene W, Alnizami H, Moses W, Morrison D. Interacting with public policy: Driving transportation policy through technological innovation. Interactions. 2010;17: 42–48. 10.1145/1806491.1806502

[14] Beckers N, Schreiner S, Bertrand P, Mehler B, Reimer B. Comparing the demands of destination entry using Google Glass and the Samsung Galaxy S4 during simulated driving. Appl Ergon. 2017;58: 25–34. 10.1016/j.apergo.2016.05.005 27633195

[15] He J, Chaparro A, Nguyen B, Burge RJ, Crandall J, Chaparro B, et al Texting while driving: Is speech-based text entry less risky than handheld text entry? Accid Anal Prev. 2014;72: 287–295. 10.1016/j.aap.2014.07.014 25089769

[16] Tippey KG, Sivaraj E, Ferris TK. Driving while interacting with Google Glass: Investigating the combined effect of head-up display and hands-free input on driving safety and multitask performance. Hum Factors. 2017;59: 671–688. 10.1177/0018720817691406 28186420

[17] Creaser JI, Edwards CJ, Morris NL, Donath M. Are cellular phone blocking applications effective for novice teen drivers? J Safety Res. 2015;54: 75–82. 10.1016/j.jsr.2015.06.014 26403905

[18] Delgado MK, McDonald CC, Winston FK, Halpern SD, Buttenheim AM, Setubal C, et al Attitudes on technological, social, and behavioral economic strategies to reduce cellphone use among teens while driving. Traffic Inj Prev. 2018; Advance online publication. 10.1080/15389588.2018.1458100 29652523 PMC6215497

[19] National Highway Traffic Safety Administration. U.S. DOT and NHTSA kick off 5th annual U Drive. U Text. U Pay. campaign [Internet]. 2018. Available: https://www.nhtsa.gov/press-releases/us-dot-and-nhtsa-kick-5th-annual-u-drive-u-text-u-pay-campaign

[20] AT&T. It Can Wait [Internet]. 2014. Available: https://www.att.com/Common/about_us/txting_driving/att_twd_fact_sheet0512.pdf

[21] Cismaru M, Nimegeers K. “Keep your eyes up, don’t text and drive”: A review of anti-texting while driving campaigns’ recommendations. Int Rev Public Nonprofit Mark. 2017;14: 113–135. 10.1007/s12208-016-0166-7

[22] Chaudhary NK, Connolly J, Tison J, Solomon M, Elliott K. Evaluation of the NHTSA distracted driving high-visibility enforcement demonstration projects in California and Delaware. (Report No. DOT HS 812 108). Washington, DC: National Highway Traffic Safety Administration; 2015.

[23] Joseph B, Zangbar B, Bains S, Kulvatunyou N, Khalil M, Mahmoud D, et al Injury prevention programs against distracted driving: Are they effective? Traffic Inj Prev. 2016;17: 460–464. 10.1080/15389588.2015.1116042 26760495

[24] Miranda B, Jere C, Alharbi O, Lakshmi S, Khouja Y, Chatterjee S. Examining the efficacy of a persuasive technology package in reducing texting and driving behavior Persuasive Technology. Springer, Berlin, Heidelberg; 2013 pp. 137–148. 10.1007/978-3-642-37157-8_17

[25] Rohl A, Eriksson S, Metcalf D. Evaluating the effectiveness of a front windshield sticker reminder in reducing texting while driving in young adults. Cureus. 2016;8: e691 10.7759/cureus.691 27555989 PMC4980211

[26] Dillard JP, Plotnick CA, Godbold LC, Freimuth VS, Edgar T. The multiple affective outcomes of AIDS PSAs: Fear appeals do more than scare people. Commun Res. 1996;23: 44–72. 10.1177/009365096023001002

[27] Maddux JE, Rogers RW. Protection motivation and self-efficacy: A revised theory of fear appeals and attitude change. J Exp Soc Psychol. 1983;19: 469–479. 10.1016/0022-1031(83)90023-9

[28] Lennon R, Rentfro R, O’Leary B. Social marketing and distracted driving behaviors among young adults: The effectiveness of fear appeals. Acad Mark Stud J. 2010;14: 95–113.

[29] King KW, Reid LN. Fear arousing anti-drinking and driving PSAs: Do physical injury threats influence young adults? Curr Issues Res Advert. 1990;12: 155–175. 10.1080/01633392.1990.10504950

[30] Ben-Ari OT, Florian V, Mikulincer M. The impact of mortality salience on reckless driving: A test of terror management mechanisms. J Pers Soc Psychol. 1999;76: 35–45. 10.1037/0022-3514.76.1.35 9972551

[31] Lewis I, Watson B, Tay R, White KM. The role of fear appeals in improving driver safety: A review of the effectiveness of fear-arousing (threat) appeals in road safety advertising. Int J Behav Consult Ther. 2007;3: 203–222. 10.1037/h0100799

[32] Carey RN, McDermott DT, Sarma KM. The impact of threat appeals on fear arousal and driver behavior: A meta-analysis of experimental research 1990–2011. PLOS ONE. 2013;8: e62821 10.1371/journal.pone.0062821 23690955 PMC3656854

[33] Kareklas I, Muehling DD. Addressing the texting and driving epidemic: Mortality salience priming effects on attitudes and behavioral intentions. J Consum Aff. 2014;48: 223–250. 10.1111/joca.12039

[34] Carey RN, Sarma KM. Threat appeals in health communication: Messages that elicit fear and enhance perceived efficacy positively impact on young male drivers. BMC Public Health. 2016;16 10.1186/s12889-016-3227-2 27460475 PMC4962518

[35] Glasman LR, Albarracín D. Forming attitudes that predict future behavior: A meta-analysis of the attitude–behavior relation. Psychol Bull. 2006;132: 778–822. 10.1037/0033-2909.132.5.778 16910754 PMC4815429

[36] Webb TL, Sheeran P. Does changing behavioral intentions engender behavior change? A meta-analysis of the experimental evidence. Psychol Bull. 2006;132: 249–268. 10.1037/0033-2909.132.2.249 16536643

[37] Harrison MA. College students’ prevalence and perceptions of text messaging while driving. Accid Anal Prev. 2011;43: 1516–1520. 10.1016/j.aap.2011.03.003 21545885

[38] Hayashi Y, Russo CT, Wirth O. Texting while driving as impulsive choice: A behavioral economic analysis. Accid Anal Prev. 2015;83: 182–189. 10.1016/j.aap.2015.07.025 26280804 PMC4604567

[39] Nemme HE, White KM. Texting while driving: Psychosocial influences on young people’s texting intentions and behaviour. Accid Anal Prev. 2010;42: 1257–1265. 10.1016/j.aap.2010.01.019 20441840

[40] Bayer JB, Campbell SW. Texting while driving on automatic: Considering the frequency-independent side of habit. Comput Hum Behav. 2012;28: 2083–2090. 10.1016/j.chb.2012.06.012

[41] Panek ET, Bayer JB, Cin SD, Campbell SW. Automaticity, mindfulness, and self-control as predictors of dangerous texting behavior. Mob Media Commun. 2015; 2050157915576046. 10.1177/2050157915576046

[42] Hayashi Y, Foreman AM, Friedel JE, Wirth O. Executive function and dangerous driving behaviors in young drivers. Transp Res Part F Traffic Psychol Behav. 2018;52: 51–61. 10.1016/j.trf.2017.11.007 PMC6477690 31024220

[43] Hayashi Y, Rivera EA, Modico JG, Foreman AM, Wirth O. Texting while driving, executive function, and impulsivity in college students. Accid Anal Prev. 2017;102: 72–80. 10.1016/j.aap.2017.02.016 28267655 PMC6481653

[44] Pope CN, Bell TR, Stavrinos D. Mechanisms behind distracted driving behavior: The role of age and executive function in the engagement of distracted driving. Accid Anal Prev. 2017;98: 123–129. 10.1016/j.aap.2016.09.030 27716494 PMC5167635

[45] Bechara A. Decision making, impulse control and loss of willpower to resist drugs: A neurocognitive perspective. Nat Neurosci. 2005;8: 1458–1463. 10.1038/nn1584 16251988

[46] Bickel WK, Johnson MW, Koffarnus MN, MacKillop J, Murphy JG. The behavioral economics of substance use disorders: Reinforcement pathologies and their repair. Annu Rev Clin Psychol. 2014;10: 641–677. 10.1146/annurev-clinpsy-032813-153724 24679180 PMC4501268

[47] Green L, Myerson J. A discounting framework for choice with delayed and probabilistic rewards. Psychol Bull. 2004;130: 769–792. 10.1037/0033-2909.130.5.769 15367080 PMC1382186

[48] Lamb RJ, Ginsburg BC. Addiction as a BAD, a Behavioral Allocation Disorder. Pharmacol Biochem Behav. 2017; Advance online publication. 10.1016/j.pbb.2017.05.002 28476485 PMC6089073

[49] Reynolds B, Schiffbauer RM. Impulsive choice and workplace safety: A new area of inquiry for research in occupational settings. Behav Anal. 2004;27: 239–246. 22478432 10.1007/BF03393183 PMC2755405

[50] Hayashi Y, Fessler HJ, Friedel JE, Foreman AM, Wirth O. The roles of delay and probability discounting in texting while driving: Toward the development of a translational scientific program. J Exp Anal Behav. 2018;Advance online publication. 10.1002/jeab.460 PMC6376405 30028007

[51] Hayashi Y, Miller K, Foreman AM, Wirth O. A behavioral economic analysis of texting while driving: Delay discounting processes. Accid Anal Prev. 2016;97: 132–140. 10.1016/j.aap.2016.08.028 27614547 PMC5154926

[52] Metcalfe J, Mischel W. A hot/cool-system analysis of delay of gratification: Dynamics of willpower. Psychol Rev. 1999;106: 3–19. 10197361 10.1037/0033-295x.106.1.3

[53] Kahneman D. Thinking, fast and slow. New York, NY: Farrar, Straus and Giroux; 2011.

[54] Bickel WK, Jarmolowicz DP, Mueller ET, Gatchalian KM, McClure SM. Are executive function and impulsivity antipodes? A conceptual reconstruction with special reference to addiction. Psychopharmacology (Berl). 2012;221: 361–387. 10.1007/s00213-012-2689-x 22441659 PMC4035182

[55] Bickel WK, Miller ML, Yi R, Kowal BP, Lindquist DM, Pitcock JA. Behavioral and neuroeconomics of drug addiction: Competing neural systems and temporal discounting processes. Drug Alcohol Depend. 2007;90: S85–S91. 10.1016/j.drugalcdep.2006.09.016 17101239 PMC2033431

[56] Mostafa MM. Neural correlates of fear appeal in advertising: An fMRI analysis. J Mark Commun. 2018; Advance online publication. 10.1080/13527266.2018.1497680

[57] Wang A-L, Ruparel K, Loughead JW, Strasser AA, Blady SJ, Lynch KG, et al Content matters: Neuroimaging investigation of brain and behavioral impact of televised anti-tobacco public service announcements. J Neurosci Off J Soc Neurosci. 2013;33: 7420–7427. 10.1523/JNEUROSCI.3840-12.2013 23616548 PMC3773220

[58] Falk EB, O’Donnell MB, Tompson S, Gonzalez R, Dal Cin S, Strecher V, et al Functional brain imaging predicts public health campaign success. Soc Cogn Affect Neurosci. 2016;11: 204–214. 10.1093/scan/nsv108 26400858 PMC4733336

[59] Posner MI, Rothbart MK, Sheese BE, Tang Y. The anterior cingulate gyrus and the mechanism of self-regulation. Cogn Affect Behav Neurosci. 2007;7: 391–395. 18189012 10.3758/cabn.7.4.391

[60] Bechara A, Damasio AR. The somatic marker hypothesis: A neural theory of economic decision. Games Econ Behav. 2005;52: 336–372. 10.1016/j.geb.2004.06.010

[61] Tannenbaum MB, Hepler J, Zimmerman RS, Saul L, Jacobs S, Wilson K, et al Appealing to fear: A meta-analysis of fear appeal effectiveness and theories. Psychol Bull. 2015;141: 1178–1204. 10.1037/a0039729 26501228 PMC5789790

[62] Koch EJ. How does anticipated regret influence health and safety decisions? A literature review. Basic Appl Soc Psychol. 2014;36: 397–412. 10.1080/01973533.2014.935379

[63] Zeelenberg M, Pieters R. A theory of regret regulation 1.0. J Consum Psychol. 2007;17: 3–18. 10.1207/s15327663jcp1701_3 PMC2435165 18568095

[64] Brewer NT, DeFrank JT, Gilkey MB. Anticipated regret and health behavior: A meta-analysis. Health Psychol Off J Div Health Psychol Am Psychol Assoc. 2016;35: 1264–1275. 10.1037/hea0000294 27607136 PMC5408743

[65] Ellis EM, Elwyn G, Nelson WL, Scalia P, Kobrin SC, Ferrer RA. Interventions to engage affective forecasting in health-related decision making: A meta-analysis. Ann Behav Med Publ Soc Behav Med. 2018;52: 157–174. 10.1093/abm/kax024 29538630 PMC7189982

[66] Quisenberry AJ, Eddy CR, Patterson DL, Franck CT, Bickel WK. Regret expression and social learning increases delay to sexual gratification. PLOS ONE. 2015;10: e0135977 10.1371/journal.pone.0135977 26280349 PMC4539230

[67] Strong JT, Anderson RE, Dubas KM. Marketing threat appeals: A conceptual framework and implications for practitioners. J Manag Issues. 1993;5: 532–546.

[68] Myerson J, Green L, Warusawitharana M. Area under the curve as a measure of discounting. J Exp Anal Behav. 2001;76: 235–243. 10.1901/jeab.2001.76-235 11599641 PMC1284836

[69] Hayes AF. Introduction to mediation, moderation, and conditional process analysis: A regression-based approach [Internet]. 2nd ed. New York, NY: Guilford Press; 2018 Available: https://www.guilford.com/books/Introduction-to-Mediation-Moderation-and-Conditional-Process-Analysis/Andrew-Hayes/9781462534654/reviews

[70] Boneau CA. The effects of violations of assumptions underlying the test. Psychol Bull. 1960;57: 49–64. 13802482 10.1037/h0041412

[71] Stonehouse JM, Forrester GJ. Robustness of the t and U tests under combined assumption violations. J Appl Stat. 1998;25: 63–74. 10.1080/02664769823304

[72] Lumley T, Diehr P, Emerson S, Chen L. The importance of the normality assumption in large public health data sets. Annu Rev Public Health. 2002;23: 151–169. 10.1146/annurev.publhealth.23.100901.140546 11910059

[73] Elliott MA. Testing the capacity within an extended theory of planned behaviour to reduce the commission of driving violations. Transportmetrica. 2012;8: 321–343. 10.1080/18128602.2010.502548

[74] Parker D, Manstead ASR, Stradling SG. Extending the theory of planned behaviour: The role of personal norm. Br J Soc Psychol. 1995;34: 127–138. 10.1111/j.2044-8309.1995.tb01053.x

[75] Chorlton K, Conner M, Jamson S. Identifying the psychological determinants of risky riding: An application of an extended Theory of Planned Behaviour. Accid Anal Prev. 2012;49: 142–153. 10.1016/j.aap.2011.07.003 23036391

[76] Newnam S, Watson B, Murray W. Factors predicting intentions to speed in a work and personal vehicle. Transp Res Part F Traffic Psychol Behav. 2004;7: 287–300. 10.1016/j.trf.2004.09.005

[77] Elliott MA, Thomson JA. The social cognitive determinants of offending drivers’ speeding behaviour. Accid Anal Prev. 2010;42: 1595–1605. 10.1016/j.aap.2010.03.018 20728608

[78] Elliott MA, Thomson JA, Robertson K, Stephenson C, Wicks J. Evidence that changes in social cognitions predict changes in self-reported driver behavior: Causal analyses of two-wave panel data. Accid Anal Prev. 2013;50: 905–916. 10.1016/j.aap.2012.07.017 22878143

[79] Gauld CS, Lewis I, White KM, Fleiter JJ, Watson B. Smartphone use while driving: What factors predict young drivers’ intentions to initiate, read, and respond to social interactive technology? Comput Hum Behav. 2017;76: 174–183. 10.1016/j.chb.2017.07.023

[80] Gauld CS, Lewis I, White KM. Concealing their communication: Exploring psychosocial predictors of young drivers’ intentions and engagement in concealed texting. Accid Anal Prev. 2014;62: 285–293. 10.1016/j.aap.2013.10.016 24211560

[81] Parker D, Stradling SG, Manstead ASR. Modifying beliefs and attitudes to exceeding the speed limit: An intervention study based on the theory of planned behavior. J Appl Soc Psychol. 1996;26: 1–19. 10.1111/j.1559-1816.1996.tb01835.x

[82] Witte K. Putting the fear back into fear appeals: The extended parallel process model. Commun Monogr. 1992;59: 329–349. 10.1080/03637759209376276

[83] Witte K. Fear as motivator, fear as inhibitor: Using the extended parallel process model to explain fear appeal successes and failures In: Andersen PA, Guerrero LK, editors. Handbook of Communication and Emotion. San Diego, CA: Academic Press; 1996 pp. 423–450. 10.1016/B978-012057770-5/50018-7

[84] Rhodes N. Fear-appeal messages: Message processing and affective attitudes. Commun Res. 2017;44: 952–975. 10.1177/0093650214565916

[85] Witte K, Meyer G, Martell D. Effective health risk messages: A step-by-step guide. Thousand Oaks, CA: Sage Publications; 2001.

[86] Lewis IM, Watson B, White KM. Response efficacy: The key to minimizing rejection and maximizing acceptance of emotion-based anti-speeding messages. Accid Anal Prev. 2010;42: 459–467. 10.1016/j.aap.2009.09.008 20159067

[87] Lang A. The limited capacity model of mediated message processing. J Commun. 2000;50: 46–70. 10.1111/j.1460-2466.2000.tb02833.x

[88] Baker F, Johnson MW, Bickel WK. Delay discounting in current and never-before cigarette smokers: Similarities and differences across commodity, sign, and magnitude. J Abnorm Psychol. 2003;112: 382–392. 10.1037/0021-843X.112.3.382 12943017

[89] Bickel WK, Pitcock JA, Yi R, Angtuaco EJ. Congruence of BOLD response across intertemporal choice conditions: Fictive and real money gains and losses. J Neurosci. 2009;29: 8839–8846. 10.1523/JNEUROSCI.5319-08.2009 19587291 PMC2749994

[90] Johnson MW, Bickel WK. Within-subject comparison of real and hypothetical money rewards in delay discounting. J Exp Anal Behav. 2002;77: 129–146. 10.1901/jeab.2002.77-129 11936247 PMC1284852

[91] Johnson MW, Bickel WK, Baker F. Moderate drug use and delay discounting: A comparison of heavy, light, and never smokers. Exp Clin Psychopharmacol. 2007;15: 187–194. 10.1037/1064-1297.15.2.187 17469942

[92] Lagorio CH, Madden GJ. Delay discounting of real and hypothetical rewards III: Steady-state assessments, forced-choice trials, and all real rewards. Behav Processes. 2005;69: 173–187. 10.1016/j.beproc.2005.02.003 15845306

[93] Madden GJ, Raiff BR, Lagorio CH, Begotka AM, Mueller AM, Hehli DJ, et al Delay discounting of potentially real and hypothetical rewards: II. Between- and within-subject comparisons. Exp Clin Psychopharmacol. 2004;12: 251–261. 10.1037/1064-1297.12.4.251 15571442

[94] Madden GJ, Begotka AM, Raiff BR, Kastern LL. Delay discounting of real and hypothetical rewards. Exp Clin Psychopharmacol. 2003;11: 139–145. 10.1037/1064-1297.11.2.139 12755458

[95] Witte K, Allen M. A meta-analysis of fear appeals: Implications for effective public health campaigns. Health Educ Behav Off Publ Soc Public Health Educ. 2000;27: 591–615. 10.1177/109019810002700506 11009129

[96] Dixon MR, Jacobs EA, Sanders S. Contextual control of delay discounting by pathological gamblers. J Appl Behav Anal. 2006;39: 413–422. 10.1901/jaba.2006.173-05 17236338 PMC1702333

[97] Van den Bergh B, Dewitte S, Warlop L. Bikinis instigate generalized impatience in intertemporal choice. J Consum Res. 2008;35: 85–97. 10.1086/525505

[98] Wilson M, Daly M. Do pretty women inspire men to discount the future? Proc Biol Sci. 2004;271: S177–S179. 10.1098/rsbl.2003.0134 15252976 PMC1810021

[99] Hayashi Y, Friedel JE, Foreman AM, Wirth O. A behavioral economic analysis of demand for texting while driving. Manuscr Submitt Publ 2018; 10.1007/s40732-019-00341-w PMC6423545 30899125

[100] Kelley NJ, Schmeichel BJ. Thinking about death reduces delay discounting. PLOS ONE. 2015;10: e0144228 10.1371/journal.pone.0144228 26630664 PMC4668029

[101] Bickel WK, Stein JS, Moody LN, Snider SE, Mellis AM, Quisenberry AJ. Toward narrative theory: Interventions for reinforcer pathology in health behavior In: Stevens JR, editor. Impulsivity. Springer International Publishing; 2017 pp. 227–267. 10.1007/978-3-319-51721-6_8 30351565

[102] Bickel WK, Yi R, Landes RD, Hill PF, Baxter C. Remember the future: Working memory training decreases delay discounting among stimulant addicts. Biol Psychiatry. 2011;69: 260–265. 10.1016/j.biopsych.2010.08.017 20965498 PMC3015021

[103] Hendrickson KL, Rasmussen EB. Effects of mindful eating training on delay and probability discounting for food and money in obese and healthy-weight individuals. Behav Res Ther. 2013;51: 399–409. 10.1016/j.brat.2013.04.002 23685325

[104] Morrison KL, Madden GJ, Odum AL, Friedel JE, Twohig MP. Altering impulsive decision making with an acceptance-based procedure. Behav Ther. 2014;45: 630–639. 10.1016/j.beth.2014.01.001 25022774 PMC5661961

[105] Yi R, Johnson MW, Giordano LA, Landes RD, Badger GJ, Bickel WK. The effects of reduced cigarette smoking on discounting future rewards: An initial evaluation. Psychol Rec. 2008;58: 163–174. 23825867 10.1007/bf03395609 PMC3697871

[106] Landes RD, Christensen DR, Bickel WK. Delay discounting decreases in those completing treatment for opioid dependence. Exp Clin Psychopharmacol. 2012;20: 302–309. 10.1037/a0027391 22369670 PMC3972253

