# Supplementary material for: Threat appeals reduce impulsive decision making associated with texting while driving: A behavioral economic approach
Source: PLoS One. 2019 Mar 7;14(3):e0213453. doi: 10.1371/journal.pone.0213453 (PMC6405105; doi:10.1371/journal.pone.0213453)
Supplement: S1 Appendix — (DOCX) [file pone.0213453.s001.docx]

**S1 Appendix. Questionnaires for TWD-related measures and manipulation check**

Anticipated regret

- If I send/read a text message while driving in the next week, I would feel regret.
  (1: S*trongly disagree*; 7: *Strongly agree*)
- If I send/read a text message while driving in the next week, I would feel sorry.
  (1: *Strongly disagree*; 7: *Strongly agree*)

Attitude

- For me, sending/reading a text message while driving in the next week would be:
  (1: *Bad*; 7: *Good)*
- For me, sending/reading a text message while driving in the next week would be:
  (1: *Worthless*; 7: *Valuable)*
- For me, sending/reading a text message while driving in the next week would be:
  (1: *Unwise*; 7: *Wise)*
- For me, sending/reading a text message while driving in the next week would be:
  (1: *Negative*; 7: *Positive)*

Intention

- I plan to send/read a text message while driving in the next week.
  (1: *Strongly disagree*; 7: *Strongly agree*)
- I intend to send/read a text message while driving in the next week.
  (1: *Strongly disagree*; 7: *Strongly agree*)
- It is likely that I will send/read a text message while driving in the next week.
  (1: *Strongly disagree*; 7: *Strongly agree*)

Perceived efficacy (control over TWD)

- I have complete control over whether I will send/read a text message while driving in the next week.
  (1: *Strongly disagree*; 7: *Strongly agree*)
- It is mostly up to me whether I will send/read a text message while driving at any speed in the next week.
  (1: *Strongly disagree*; 7: *Strongly agree*)

Past TWD frequency

- In the past week, how often did you send a text message while driving?
  (1: *Not at all*; 7: *Always*)
- In the past week, how often did you read a text message while driving?
  (1: *Not at all*; 7: *Always*)

Perceived threat (manipulation check)

- To what extent did the video that you watched make you think of potentially killing someone if you text while driving?
  (1: *Not at all*; 7: *Extremely*)
- Please indicate your level of agreement with the following statement: The video that I watched reminds me of a possibility of killing someone if I text while driving.
  (1: *Strongly disagree*; 7: *Strongly agree*)
- Please indicate your level of agreement with the following statement: The video that I watched invokes thoughts of potentially killing someone if I text while driving.
  (1: *Strongly disagree*; 7: *Strongly agree*)
